# Ultrasound diagnosis of cephalopagus conjoined twin pregnancy at 29 weeks

**DOI:** 10.2349/biij.6.4.e38

**Published:** 2010-10-01

**Authors:** D Sabih, E Ahmad, A Sabih, Q Sabih

**Affiliations:** 1 Multan Institute of Nuclear Medicine and Radiotherapy, Multan, Pakistan; 2 City Hospital, Multan, Pakistan; 3 Aga Khan Medical University, Karachi, Pakistan; 4 UNM School of Medicine, Albuquerque, USA

**Keywords:** conjoined twins, ultrasound, missed diagnosis, false negative, cephalopagus

## Abstract

The authors report a case of a cephalopagus conjoined twin that was diagnosed at 29 weeks of gestation despite the mother having had two ultrasounds done previously. The fetus had one head and face, fused thoraces, common umbilicus but had two pelvises and two sets of genitalia. The fetus had four normally formed legs and arms.

Antenatal ultrasound images are supplemented by post natal photographs. A review of literature, clues to ultrasound diagnosis and possible causes of missing this significant abnormality until the 3rd trimester are discussed.

## INTRODUCTION

Conjoined twinning is a rare aberration of monozygotic monoamniotic twinning and results in fusion of the twins at any part of their body. Conjoined twinning is usually thought to occur with late division (post day-13) of the embryonic disc and “conjoined” might actually be a misnomer since it is a failure of fission. Although, some authors have proposed that conjoined twins occur from secondary fusion of two separate embryos [1]; the fusion theory has, however, been largely discarded [2]. The site, type and extent of joining is infinitely variable [3] giving rise to a complex nomenclature, a variable prognosis and varying outcomes of attempted surgical separation.

Conjoined twins are colloquially called Siamese twins; the original “Siamese” twins Chang and Eng were actually born in Siam (present day Thailand) in 1811, lived for 62 years, travelled widely and emigrated to the US; they worked as exhibits in circuses, were subjects of scientific research and tilled the land as farmers; they married separate women (who were sisters) and fathered 22 children between them [4]. The “Siamese” twins are not the oldest known cases and the first named twins to reach adulthood might be the Biddenden maids Mary and Eliza Chulkhurst born in 1100 AD, though the veracity of their existence has been doubted [5].

Some conjoined twins live to adulthood and live seemingly ordinary lives [6], but most die *in utero* (about 65%) or within the first 24 hours [7]; the rare ones that do survive become the subject of fascination due to their dramatic nonconformity of anatomy. Conjoined twins are frequently mentioned in mythology as well as ancient history.

**Figure 1:**
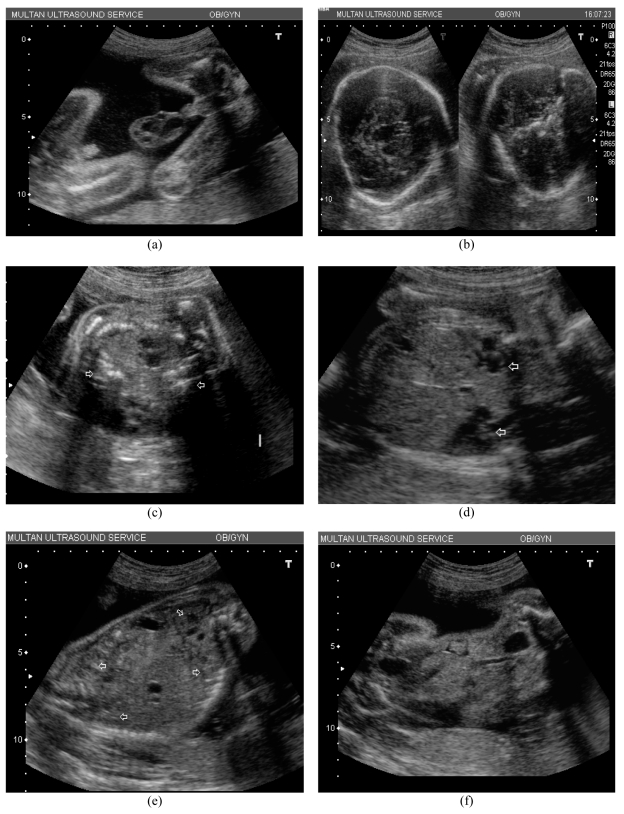
a) Transverse ultrasound sections of the fetal head; note delta shape (left) and absence of cerebellum and posterior fossa (right); b) transverse section of the fetal thorax, note two spines on opposite sides of a fuse thorax, arrows mark vertebrae; c) transverse section of the fetal thorax, showing two unfused hearts (arrows); d) transverse section of the upper abdomen, four kidneys are seen (arrows); e) an oblique transverse section of the fetal abdomen, note the two bladders, the way the fetal lower abdomens angulate and the obliquity of the section, this is a relatively coronal section and the fused liver and unfused bladder are seen in both fetuses; f) the two pelvises and external genitalia and four thighs are seen as the lower unfused abdomens diverge; a cross section of the cord shows multiple vessels.

## CASE REPORT

A 28-year-old healthy woman, gravida 2, para 1, came for a routine ultrasound at 29 weeks of gestation; her previous pregnancy had been uneventful and the child was alive and healthy. There was no consanguinity and no history of twinning in the family. Ultrasound was done with a digital scanner using a broad-band convex transducer (Toshiba, SSA-550A; with PVM375-AT probe; Toshiba Medical Systems, Tustin CA).

The ultrasound showed a fetus with an unusual delta-shaped head in transverse section, the cerebellum could not be seen, there was a fused thorax showing two spines, two separately beating hearts, two rib cages with sharing of the liver across the fetuses, there were four kidneys and two bladders; the lower abdomens were separate with two male external genitalia and four legs; and the cord had multiple vessels (Figure 1).

The parents opted for a termination and a cephalopagic fetus with the abnormalities noted on ultrasound was delivered. The head had an unusual broad outline but the vertex seemed normal and the face seemed normal too. There were two ears. A small complex irregular mass with multiple ridges was seen over the occipital midline.

The parents did not give permission for an autopsy so the internal anatomy could not be confirmed.

## DISCUSSION

Conjoined twins are rare and are reported to occur with a prevalence of 1:50,000 *in utero* to 1:250,000 live births [8]. The actual rates might be higher because of unreported termination. Conjoined twins are generally incompatible with life i.e. 65% of cases are stillborn while of those that are born alive, 35% die within the first 24 hours [7]. Only 25% survive to an age where surgical separation can be considered [9]. The incidence of monozygotic twinning is independent of race, maternal age and geography. There is a predominance of females in conjoined twins on the order of 3:1 and no increase in risk is known with parity, race, maternal age or heredity [10]. Over 1,000 descendants of the original “Siamese” twins were studied; the next twinning took place after the 4th generation, these were normal monozygotic healthy twins [11], this supports the absence of hereditary influence on conjoined twinning.

Conjoined twinning can occur at infinite sites of junction [3] giving rise to a complex and sometimes confusing classification [12]. Usually, the same sites fuse but parasitic twins are a subset where “asymmetric” joining occurs [2]. Parasitic twins have their own variants and independent classification [13]. The conjoined twins, though monozygotic, are less “identical” than the usual, separate identical twins; one twin might have more internal disarray of anatomy than the other [14, 15].

Conjoined twins are classified according to the site of fusion. When there is complete duplication, the region of duplication (usually with ventral fusion) is used to name the class, followed by the Greek term for fastened “pagus” (e.g. thoracopagus: joined at the thorax, craniopagus: joined at the head). When the duplication is incomplete, the fusion is more extensive and usually lateral; the part that remains duplicate and separate denotes the type of conjoining e.g. dicephalus: two heads, dipygus: two pelvises, etc; dorsal union (rachipagus) is extremely rare [3, 12, 16, 17]. The case reported here has been called cephalothoracopagus [2] or cephalopagus [12].

**Figure 2:**
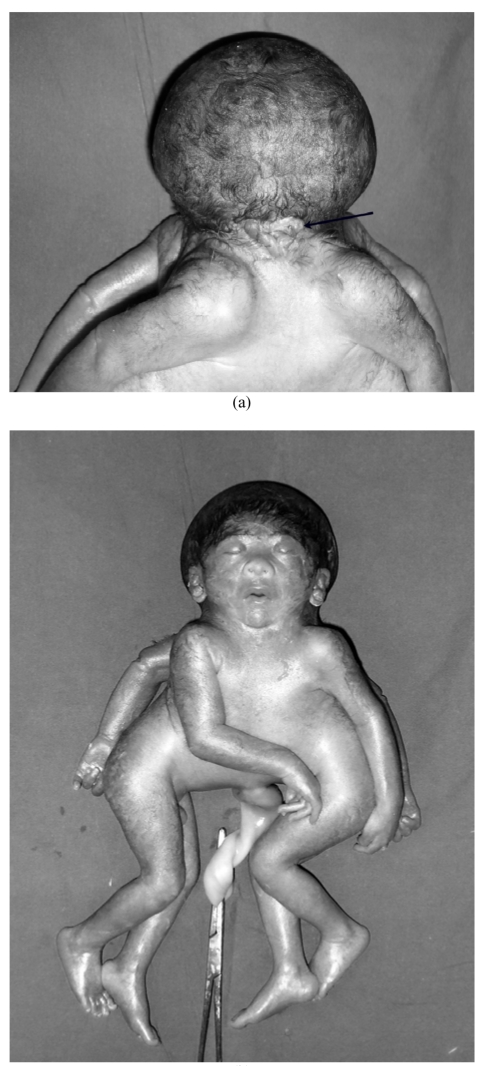
a) Anterior photograph of the delivered fetus, the face and head look almost normal; b) posterior photograph of the delivered fetus, arrow points to a small ridged structure over the occiput which is probably the otocephalic face on the other side of the “normal” face.

Conjoined twins with sharing of the neural structures are very rare (11%) [2] and include the following types: craniopagus, with various grades of skull, meningial and dural venous sinus sharing; there is no sharing of the face, foramen magnum or brain. Although the brain commonly remains separate, it may be connected by a bridge of neural tissue and the trunks are not joined [14]. Classifications concerned with surgical separation divides craniopagus twins into partial and complete forms depending upon the degree of dural venous sinus sharing [18] or the region of conjunction (frontal, parietal, temporoparietal and occipital) [19]. Syncephalus, involves fusion of the brain and varying degree of facial fusion also. Cephalothoracopagus or cephalopagus involves the brain, face, thorax and upper abdomen; this could either be the Janiceps (Greek god with two faces) type, with two faces on either side of the head; or more rarely, one side of the head is relatively normal with a small otocephalic face on the other side (Figure 2b). Klinkosch [20] made beautiful engravings of this type of twinning in his book published in 1767, these have been reproduced by Kaufman [2]. The engraving of the base of the brain shows one cerebrum but two obliquely oriented diverging cerebella and brain-stems; these would explain the inability of cerebellar visualization in the expected location within the posterior fossa on ultrasound in the authors' case. The heart and liver are often conjoined in cephalopagi, but in their case there were two separate hearts, although the liver was shared by the twins.

Before the widespread use of ultrasound, most conjoined twins could not be identified *in utero*. Even with radiographs, only about 25% of cases could be diagnosed in one series [3]. Plain X-rays were often not helpful because diagnosis could only be made if there was bony fusion. Contrast radiography could be used with the contrast material instilled in the amniotic cavity in the hope of outlining the skin surface enabling continuity to be seen. However, the presence of polyhydramnios usually diluted the contrast and the procedure’s information content. The first case diagnosed primarily by ultrasound was reported in 1977 [21] at 28 weeks of gestation. Since then, criteria for ultrasound diagnosis have been well established (Table 1). There are also known pitfalls, which if avoided, will make the diagnosis feasible in most cases. First trimester ultrasound diagnosis is possible [22, 23] and the earliest diagnosis was reported after about seven weeks of pregnancy [24]. However, first trimester ultrasound diagnosis requires care and follow-ups to confirm the initial impression [25, 26].

**Table 1:** Ultrasound criteria for diagnosis of conjoined twinning (adapted from [3, 7, 19, 24, 25, 26])

**Clues**	**Pitfalls**
Bifid appearance of the 1^st^ trimester fetal pole with a “V” or “Y” shaped embryo	
Continuous skin contour at the same anatomic level	
Single amniotic cavity with no dividing membrane	In some diamniotic twins the dividing membrane can be very thin and difficult to identify
Single placenta	
Fetal anomalies like omphaloceles, complex cardiac malformations etc	The conjoining might be so severe as to mimic a single fetus with multiple anomalies
Abnormal number of vessels (more than 3) in the cord	
Unusual extension of the spines and both fetuses facing each other	All conjoined twins do not present as mirror image fetuses and can be miss-diagnosed as a single fetus with malformation.
Bi-breech or bi-cephalic presentation	Discordant presentation is possible in omphalopagus twins where the bridge is pliable and allows rotation [35].
Single heart with gross and complex anomalies	Both fetuses might have their own hearts
Fixed position of the fetus relative to each other on multiple exams	During first trimester, before amnio-chorionic separation and with a relatively small amniotic cavity the twins might be apposed to each other, appear to be fixed and having skin continuity[24, 27]
Heads at the same level	In bi-cephalic pregnancy, one head might be fixed and the other higher up

The authors’ case was diagnosed at 29 weeks of gestation after the mother had undergone two ultrasounds at 22 weeks and at 28 weeks during which no abnormality was reported. Then she was referred to us for evaluation of mild polyhydramnios. Ultrasound diagnosis of such an obvious and gross anomaly should be straightforward and it seemed difficult to understand how such a dramatic abnormality could be missed on repeated ultrasound examinations, but such a case is not an isolated case as we reported another dicephalic fetus that went unreported until 28 weeks [27]. Others have reported a false negative diagnosis of a pyopagus tetrapus parasitic twin on three scans, being confirmed only at 28 weeks on the fourth exam [28], several other 3rd trimester diagnoses were also reported [29, 30]. It is possible that if this condition is not thought of it will be missed, similar to missing a twin on a late ultrasound or a triplet when a twin is diagnosed just because the sonologist is not thinking of the possibility [31, 32]. Ultrasound (non-panoramic) in the third trimester can only show a part of the fetal anatomy and it is possible that the structure of one fetus is ascribed to the other fetus causing a mistaken impression of normal anatomy.

This case highlights the features of an extremely rare fetal malformation and concludes with the suggestion that everyone's mind should be kept open to all possibilities when making a diagnosis, as rare causes are rare but do occur in everyday practice of medicine.
